# Protein Kinase Cɛ in the Platelet and Hippocampal Tissue as a Diagnostic Biological Marker in Alzheimer Disease

**DOI:** 10.32598/bcn.9.10.80.1

**Published:** 2019-11-01

**Authors:** Sara Amiri, Kayhan Azadmanesh, Marzieh Dehghan Shasaltaneh, Baharak Khoshkholgh-Sima, Nasser Naghdi

**Affiliations:** 1. Department of Physiology and Pharmacology, Pasteur Institute of Iran, Tehran, Iran.; 2. Department of Virology, Pasteur Institute of Iran, Tehran, Iran.; 3. Department of Biology, Faculty of Sciences, University of Zanjan, Zanjan, Iran.

**Keywords:** Alzheimer disease, PKCɛ, Platelet, Hippocampus, Amyloid Beta

## Abstract

**Introduction:**

Alzheimer Disease (AD) is a neurodegenerative disorder characterized by the progressive loss of memory and other cognitive functions. Protein Kinase Cɛ (PKCɛ) is an isoform that most effectively suppresses Amyloid Beta (Aβ) production and synaptic loss.

**Methods:**

In this study, spatial learning and memory for treated rats were evaluated by the Morris water maze test. The activity (total PKC), mRNA expression, and protein level of PKCɛ in the platelet and hippocampal tissue were evaluated using immunosorbent assay, real-time qPCR, and western blotting analysis, respectively.

**Results:**

The traveled distance was significantly prolonged, and escape latency significantly increased in Aβ-treated groups. PKC activity assay showed that there was a remarkable difference between the Aβ-treated and sham-operated groups on days 10 and 30 in the hippocampus and also day 30 in platelet after the injection of Aβ. A significant effect in PKC activity was observed between days 0 and 10, days 0 and 30, as well as days 5 and 30. Aβ significantly downregulated the PKCɛ mRNA expression in the hippocampus of rats on day 30; however, no significant difference was observed in platelet. Western blot analysis demonstrated that Aβ significantly reduced PKCɛ protein expression in the hippocampus of treated groups on day 30.

**Conclusion:**

The expression level of PKCɛ was downregulated following the injection of Aβ in the hippocampus, but no significant difference was observed between the AD and sham groups in platelet that may be due to the low concentration of PKCɛ or duration of Aβ exposure in the rat brain.

## Highlights

- Amyloid Beta (Aβ) significantly downregulated the PKCɛ mRNA expression in the hippocampus of rats on day 30.
- Aβ could not significantly downregulate the PKCɛ mRNA expression in platelet on day 30.
- Western blot analysis demonstrated that Aβ significantly reduced PKCɛ protein expression in the hippocampus of treated groups on day 30.
- The expression ratio of PKCɛ was downregulated following the injection of Aβ in the hippocampus.
- There was no significant difference between the Alzheimer and sham groups in the platelet.

## Plain Language Summary

Alzheimer Disease (AD) is one of the most important neurodegenerative disorders characterized by neurofibrillary tangles and senile plaques. Protein Kinase C (PKC) is a family of serine/threonine protein kinases that are involved in signal transduction in the central nervous system. Abnormalities in the levels and activities of protein kinase C isozymes have been reported in the brain and other tissues. PKC is one of the enzymes related to amyloid precursor protein. In the neurons of AD patients, the first abnormality is a defect in the PKC signal channels. The inhibition of PKC activity leads to the reduced capacity of learning and memory. In the present study, spatial learning and memory for treated rats were evaluated by the Morris Water Maze (MWM) test. Also, the activity, mRNA expression, and protein level of PKCɛ in the platelet and hippocampal tissue of rat brains were evaluated. According to the results, the expression of PKCɛ was downregulated following the injection of amyloid beta in the hippocampus, but no significant difference between the AD and sham groups were observed in platelet that may be due to the low concentration of PKCɛ or duration of exposure to amyloid beta in the rat brain.

## 1. Introduction

Alzheimer Disease (AD) is one of the most common neurodegenerative disorders. It is characterized by the loss of mental, behavioral, and functional abilities. Formation of amyloid plaques, tau hyperphosphorylation, and inflammation lead to synaptic impairment and destroying the integrity of brain functions (Kumar, Singh, & Ekavali, 2015). Pyramidal cells of the entorhinal cortex and the CA1 region of the hippocampus are destructed in Alzheimer brains (Huang & Mucke, 2012), which may be resulted from the deficiency of several enzymes in the mentioned regions, such as protein kinase C (PKC) and mitogen-activated protein kinase (MAPK) (Hong-Qi, Zhi-Kun, & Sheng-Di, 2012). PKC alters in fibroblasts, lymphocytes and red blood cells of AD patients; therefore, based on the previous reports, PKC conformation in peripheral cells can be an early predictive marker for AD (Humpel, 2011).

PKC isoenzymes increase during the associative learning and memory processes. Synaptogenic pathways are activated during the enhancement of PKC, which plays a critical role in associative learning and the regulation of synaptic and memory functions (Sun, Nelson, & Alkon, 2015).

An increase in the enzyme’s apparent affinity for Ca ^+2^ and membrane phospholipids leads to stimulate PKC. Diacylglycerol (DAG) is generated by receptor-mediated hydrolysis of phosphoinositides in the PKC. The stimulation of PKC by its substrate like DAG translocates the enzyme from the cytosol to the specific location of neuronal tissues (Wang, Pisano, & Friedman, 1994).

The deficits in proper PKC translocation worsen stroke outcome and amyloid beta (Aβ) toxicity. PKC isoforms or “memory kinases” contribute to cognitive decline and its alteration by aging and AD progression (Sun & Alkon, 2014). They phosphorylate several proteins in the signaling pathway, like tau protein; therefore, it is identified as tau kinase. Tau hyperphosphorylation and phosphorylation of glycogen synthase kinase 3β (GSK-3β) are considered as one of the critical functions of PKC (De Montigny, Elhiri, Allyson, Cyr, & Massicotte, 2013).

PKC α, ɛ, and δ isoenzymes are associated with the α-secretase activity, increasing the non-toxic Soluble Amyloid Precursor Protein-α (s-APPα), and indirectly reducing the toxic Aβ as the degeneration product of β-secretase-mediate cleavage of APP (Khan & Alkon, 2010). Repression of the PKC gene has been linked to impaired memory and learning and their associated neurode-generative diseases caused by several neurotransmitters, such as glutamate, dopamine, acetylcholine, and serotonin.

PKCɛ is one of the most critical isoenzymes involved in memory functions at several levels, such as post-translational modification of synaptic proteins, transcriptional activity, and local protein synthesis in synapses (Sun et al., 2015). The tertiary structure of PKCɛ includes a catalytic domain and two C1 domains with a direct translocation from the plasma membrane to the nuclear membrane. PKCɛ is associated with the regulation of memory and wound healing (Lucke-Wold et al., 2015). It is necessary for spatial memory formation and object recognition through functional reduction of Aβ accumulation and inducing the endothelin-converting enzyme to degrade Aβ40 and Aβ42 to small fragments (Pacheco-Quinto & Eckman, 2013).

The neuroprotective role of PKCɛ is through its translocation to synaptic membrane; therefore, it can be protective against memory decline in AD (Lucke-Wold et al., 2015). The loss of synaptogenic action of PKCɛ, its protective effects against neurogenic factors, lower levels of PKCɛ in the hippocampal area of AD brain are significant markers to distinguish AD from other disorders (Hongpaisan, Sun, & Alkon, 2011; Nelson, Sun, Hongpaisan, & Alkon, 2008). PKC and synaptic growth factors, like brain-derived neurotrophic factor (BDNF), are associated with synaptic regulation and replacement with a vital role at the core of the disease-modifying therapeutics (Sun et al., 2015). The high abundance of PKCɛ in presynaptic nerve fibers demonstrates a function in neurite outgrowth, synapse formation, and neurotransmitter release (Hongpaisan et al., 2011).

Phospholipid-dependent PKC activities and levels decrease in leukocytes and platelets of AD patients compared with the controls (Bosman et al., 1992; Lanius et al., 1997; Matsushima et al., 1994; Molchan et al., 1993). Low-density platelet populations reveal the increased serotonin content in dementia of the Alzheimer type (Milovanovic et al., 2014). Platelets provide APP as a precursor Aβ protein, so they are probably involved in AD pathophysiology. One of the features of progressive AD is malfunctioning platelets in dementia (Jaremo, Milovanovic, Buller, Nilsson, & Winblad, 2012). Human platelets contain high levels of APP and the concentrations of platelets are equivalent to the number of APP isoforms in the brain (Catricala, Torti, & Ricevuti, 2012).

Platelet agonists activate specific signaling pathways, such as different molecules and enzymes. Therefore, they can evoke a transient enhancement of intracellular Ca ^+2^ concentrations (Catricala et al., 2012). The metabolism of platelet APP might also contribute to the accumulation of Aβ in the brain (Deane & Zlokovic, 2007; Roher et al., 2009). In the present study, we investigated the alteration of PKCɛ in the platelet and hippocampal tissue in the pathological diseases, like AD as a biological marker to detect the primary stages of the AD.

## 2. Methods

### 2.1. Animals

Adult male Wistar rats weighting 200–230 g were purchased from the Pasteur Institute of Iran and housed 4 per cage in large cages. Food and water were available, and the animals maintained at room temperature (24±1ºC) with a 12/12 h light/dark cycle (lights on at 7:00 AM). All efforts were made to reduce the number of testing on animals and their suffering during the experiments. After a week of adaptation to the room conditions, the rats were randomly divided into three groups (n=8) of the control (intact), sham (vehicle), and experimental. The experimental group was subjected to a bilateral injection of Aβ into the CA1 region, and because no significant difference was observed between the sham and control groups, in all experiments, the sham group was compared with the Aβ-injected group.

### 2.2. Drug administration

Human Aβ (1–42) (Tocris, UK) was dissolved in sterile deionized water (vehicle) at a concentration of 5 μg/μL and incubated at 37 ^o^ C for 4 days to obtain the aggregated form. The animals were anesthetized with a combination of ketamine (50 mg/kg, IP) and xylazine (5 mg/kg, IP) and placed in a stereotaxic apparatus (Stoelting, Wood Dale, IL, USA). One microliter Aβ or vehicle (sham group) was infused bilaterally into the CA1 region of the hippocampus (1 μL/CA1) according to the rat brain atlas of Paxinos.and Watson (Paxinos & Watson, 1986) with the following coordinates: AP −3.80 mm from bregma, ML ±2.2 mm from midline, and DV −2.7 mm from the skull surface. The rate of injection was 0.5 μL/min. After completion of the infusion, the needle was left in place for 3 min to allow diffusion of the drug from the needle.

### 2.3. Behavioral assessment

The Morris Water Maze (MWM) test was performed in a water tank according to a previous procedure conducted in our laboratory. In short, animals received a block of 4 trials during 5 daily sessions. During the first 4 days (on days 6, 7, 8 and 9 after Aβ injection), the platform site did not change throughout training and situated in the center of the southwest quadrant, but animals were released randomly into the water (while facing to the tank wall) from a different location (north, east, south, and west) between the trials. The platform was submerged 1.5 cm below the surface of the water for testing spatial learning and memory. A trial was started by placing a rat into the pool. Each of four starting positions was used once in a series of four trials. Each trial was terminated as soon as the rat had climbed onto the escape platform or when 90 s had elapsed. A rat was allowed to stay on the platform for 20 s. Then, it was taken from the platform, and the next trial was started. If rats were not able to find the platform after 90 s, they would be put on the platform by the experimenter and allowed to rest on it for 20 s. At the end of the fourth trial, rats were gently dried with a towel, kept warm for an hour and returned to their home cages.

The path of each rat in each trial was automatically recorded by a computerized system and then analyzed by several parameters, such as escape latency to find the platform and traveled distance. All experiments were conducted between 10:00 AM and 1:00 PM. For each animal, two factors were evaluated: the time spent to find the platform (escape latency, s) and the distance traveled before finding the platform (cm).

Another assessment was done by visual and spatial probe test. On day 5 of behavioral assessment (ten days after Aβ injection), the platform was elevated above the water surface, covered by bright color aluminum foil, and placed in the center of the southeast quadrant, which assessed the motivation and sensorimotor coordination toward a visible platform. The spatial probe test was also performed on day 5, in which the hidden platform was removed from the target quadrant and rats were allowed to swim for 90 s. The rats were released in the water in a location that was exactly opposite from where the platform was placed. Their behavior was recorded with a video tracking system and also escape latencies were noted for further analysis.

### 2.4. Sample collection and processing

Blood samples were collected on days 0, 5, 10, and 30 after injection of Aβ or solvent and also from the control (intact) group and then the animals were decapitated. The brains were removed, and then hippocampus was separated.

### 2.5. Histological evaluation

One rat from each Aβ-injected group (10 and 30 days) was anesthetized and then transcardially perfused with phosphate-buffered saline followed by 4% (w/v) formaldehyde. Brains were rapidly removed and fixed in 30% (v/v) buffered formalin, embedded with paraffin, cut into consecutive 5-μm transverse sections with a microtome and placed on poly-d-lysine-coated glass slides. The sections were stained with Congo red to observe Aβ plaques.

### 2.6. Blood sample and platelet isolation

Rats were anesthetized with ether and blood samples were collected into a tube containing anticoagulant acid citrate dextrose. They were centrifuged immediately at 2300×g to obtain Platelet-Rich Plasma (PRP). The duration of centrifugation is associated with the volume (10 s/mL). The PRP was centrifuged at 2200×g for 8 min to obtain the platelet pellet. The plasma was removed and the platelets were resuspended in Tyrode’s buffer (10 mM HEPES, 0.4 mM NaH _2_ PO _4_ , 137 mM NaCl, 5.5 mM glucose, 2.8 mM KCl, 1 mM MgCl, and 12 mM NaHCO _3_ ) containing heparin (10 U/mL) and PGI _2_ (0.5 μM). After incubation for 10 min at 37°C, PGI _2_ (0.5 μM) was added and depending on the suspension volume, centrifuged at 1900×g. The buffer was removed and the platelets resuspended again in Tyrode’s buffer containing 0.5 μM PGI _2_ and after incubation for 10 min at 37°C, it was centrifuged for the second time. The platelet pellet was finally frozen and stored at −80°C until use.

### 2.7. The PKC activity assay

Tissue samples and platelets for PKC activity assay were prepared. The hippocampus and platelet pellet were homogenized in lysis buffer containing Tris-HCl (20 mM; pH 7.4), EGTA (2 mM), EDTA (5 mM), 0.2% Triton X-100, dithiothreitol (1 mM), protease inhibitor cocktail (P8340), and phosphatase inhibitor cocktail (P5726) (Sigma-Aldrich). Homogenates were centrifuged at 14,000 g for 10 min to remove debris. The concentration of protein was determined using the QuantiPro BCA assay kit (Sigma-Aldrich).

PKC activity was measured using a kit according to the manufacturer’s instructions (ADI-EKS-420A, Enzo Life Sciences), which is based on a solid-phase enzyme-linked immunosorbent assay. It utilizes a specific synthetic peptide as a substrate for subtypes of PKCs and a polyclonal antibody for the phosphorylated form of the substrate. The absorbance was determined at a wavelength of 450 nm, and PKC activity was expressed as a relative activity. Data were normalized to total protein content (μg), as measured by the BCA assay.

### 2.8. RNA extraction

Total RNA was extracted from the hippocampus and isolated platelets using the TRIzol reagent (Invitrogen), based on the manufacturer’s instructions. Briefly, after homogenizing by TRIzol reagent, chloroform (Merck, Germany) was added to the solution and centrifuged for 15 min at 12000×g at 4°C. The upper phase was then transferred into another tube, and RNA was precipitated with isopropanol (Merck) at 4°C. The mixture was centrifuged for 15 min at 12000×g at 4°C. The resulting pellet was then washed in 75% (v/v) ethanol and dissolved in diethylpyrocarbonate (DEPC)-treated water. The concentration of the isolated RNA was determined with the Picodrop spectrophotometer.

### 2.9. Complementary DNA (cDNA) synthesis

Complementary DNA (cDNA) synthesis was carried out using 1 μg of RNA and RevertAid RT (200 U/μL) with oligo(dT)18 (Thermo scientific) priming in a 20 μL reaction according to the manufacturer’s instructions. Rat specific primers for PKCɛ, β-actin, and Glyceraldehyde-3-Phosphate Dehydrogenase (GAPDH) were synthesized as HPLC grade. All used primers were blasted against the rat genome to ensure that they are not complementary to other regions of the genome. Table 1 presents the used primers.

**Table 1.** The sequences of the used primers

**No.**	**Gene Name**	**Primer Sequence**
1	PKCɛ-F	AAGGTGTTAGGCAAAGGCAG
	PKCɛ-R	GCAGCAATAGAGTTGGGTTAG
2	β-Actin-F	GGAGATTACTGCCCTGGCTCCTAGC
	β-Actin-R	GGCCGGACTCATCGTACTCCTGCTT
3	GAPDH-F	TGGAGTCTACTGGCGTCTT
	GAPDH-R	TGTCATATTTCTCGTGGTTCA

### 2.10. Quantitative real-time PCR (RT-qPCR)

PKCɛ expression was assessed by RT-qPCR using SYBR Premix Ex Taq II (TAKARA). For each subject, the PCR reaction was performed in triplicate in a final volume of 20 μL using 2 μL of synthesized cDNA. The PCR reactions were performed on ABI 7500 fast (Applied Biosystems) with the recommended cycling temperatures provided in the TAKARA guide.

The relative expression software tool 2009 (REST, QIAGEN) was used to compare the PKCɛ expression of the Aβ-treated and sham groups in the hippocampus and platelet samples. The expression ratio of PKCɛ was measured and the results were normalized by the GAPDH and β-actin mRNA as the reference genes. Serial dilutions were done to create a standard curve and threshold cycle numbers were averaged.

### 2.11. Quantitation of PKCɛ by Western Blotting

The hippocampus and isolated platelets were homogenized in the lysis buffer containing 1 mmol/L EDTA, 150 mmol/L NaCl, 1% (v/v) Triton X-100, 50 mmol/L Tris-HCl, 0.1% (v/v) SDS (pH 8), and protease inhibitors cocktails. Total proteins were electrophoresed in 12% SDS-PAGE gels, transferred to Polyvinylidene fluoride membranes (PVDF), incubated with anti-PKCɛ (C-15; Santa Cruz, 1/500 [v/v] and 1/1000 [v/v] dilution for platelet and hippocampus, respectively) and anti-β-actin (R-22; Santa Cruz, 1/1000 [v/v] dilution) antibodies followed by enhanced chemiluminescence detection (ECL; Amersham). Data analysis was done using Image J after background subtraction, and the densities of PKCɛ bands were measured and their ratios to β-actin were evaluated. Protein concentration was determined using the QuantiPro BCA assay kit (Sigma-Aldrich).

### 2.12. Statistical analysis

The obtained data were expressed as Mean±SEM and analyzed by One-Way Analysis of Variance (ANOVA), followed by Tukey’s posthoc test. In all comparisons, P<0.05 was used as the criterion for statistical significance. In this study, t-test was used for comparison between the sham and treated groups following the injection of Aβ on days 0, 5, 10, and 30, separately.

## 3. Results

### 3.1. Histological study

**Figure 1.**
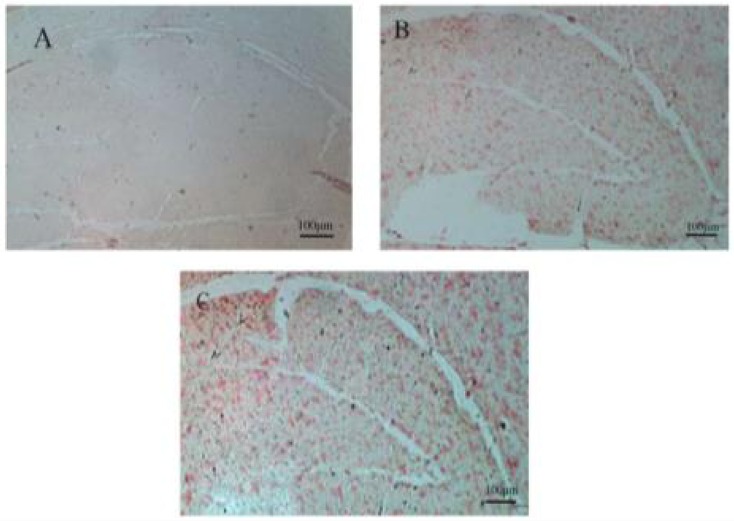
Histological observation of Amyloid Beta (Aβ) plague in the rat hippocampus using Congo red staining A. Sham group; B and C. Days 10 and 30 following the injection of Aβ in the hippocampal tissue. Red colors show the Aβ plague in the rat hippocampus.

The histological observation showed that Aβ plaques appeared in the rat brain slices after Aβ injection on days 10 and 30 (Figure 1).

### 3.2. The effect of Aβ injection on spatial memory of rats tested by the Morris water maze test

#### 3.2.1. Acquisition tests

**Figure 2.**
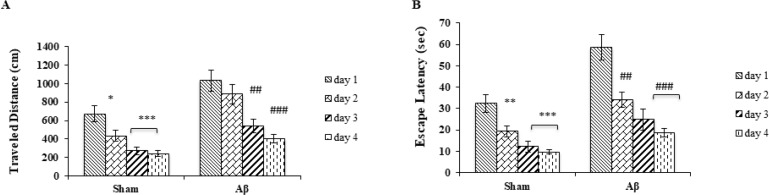
Trials performed in four consecutive days in the Morris water maze test A. Traveled distance (cm) to find the platform; and B. Escape latency (s) to find the platform Values are expressed as Mean± SEM. ^*^ P<0.05, ^**^ P<0.01, and ^***^ P<0.001 compared with the first day in the sham group; ^#^ P<0.05, ^##^ P<0.01, and ^###^ P<0.001 compared with the first day in the Aβ-treated group.

The traveled distance for finding the platform below the water surface during 4 days of acquisition trials was significantly longer in the Aβ-treated group (5 μg/μL) (F _3,28_ =10.224; P<0.001) (Figure 2 A). Escape latency significantly decreased in Aβ-treated group (F _3,28_ =13.476; P<0.001) for 4 days (Figure 2 B). There was a significant difference in terms of average escape latency (F _3,28_ =19.508) and traveled distance (F _3,28_ =13.441) in the treated group compared with the sham groups during 4 days of trials (P<0.001).

#### 3.2.2. Probe trial test

**Figure 3.**
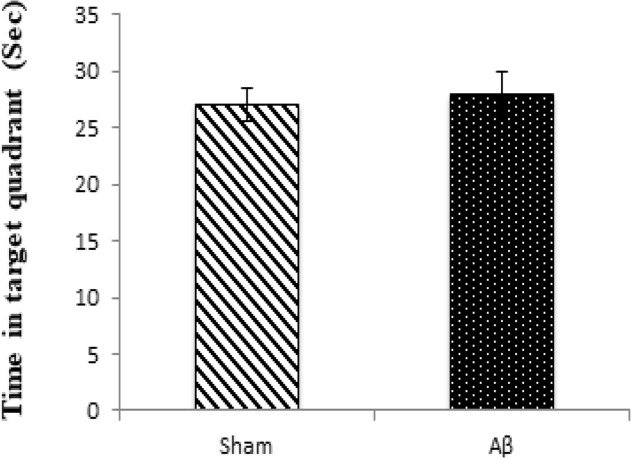
Probe test and the time spent in the target quadrant (Sec) Values are expressed as Mean±SEM.

The results of the probe trial were evaluated based on the time spent in the target quadrant. No statistically significant differences were found in probe trial compared with the sham (P>0.05) and control (P>0.05, data are not shown) groups in escape latency onto the visible platform on day 5 (Figure 3).

### 3.3. PKC activity

**Figure 4.**
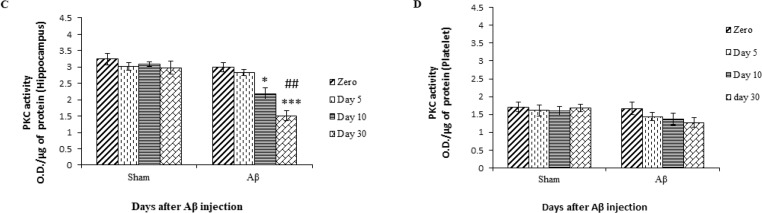
PKC activity assay at four time points (0, 5, 10, and 30 days) after Aβ injection A. Comparison between the treated and sham groups using the hippocampus. ^**^ P<0.01, and ^***^ P<0.001 compared with the sham group on days 10 and 30, respectively. B. Comparison between the treated and sham groups using platelet. ^**^ P<0.01 compared with the sham group on day 30. C. Comparison between the treated and sham groups using the hippocampus, ^*^ P<0.05 and ^***^ P< 0.001 compared with day one, and ^##^ P<0.01 versus day 5 in treated groups. D. Comparison between the treated and sham groups using platelet.

A significant difference was observed between the Aβ-treated and sham groups in terms of PKC activity in the hippocampus samples on days 10 and (P<0.01 and P<0.001, respectively) 30 following Aβ injection, but there was a significant difference in the PKC activity in the platelet sample between the Aβ-treated and sham groups on day 30 (P<0.01) after Aβ injection (Figure 4 A & B).

Also, there was a significant difference on the days 0 and 10 (F _3,28_ =19.08; P<0.05), days 0 and 30 (F _3,28_ =19.08; P<0.001), and days 5 and 30 (F _3,28_ =19.08; P<0.01) following injection of Aβ in the hippocampal tissue of the treated groups, but no significant difference was observed in platelet samples using one-way ANOVA (Figure 4 C & D).

**Figure 5.**
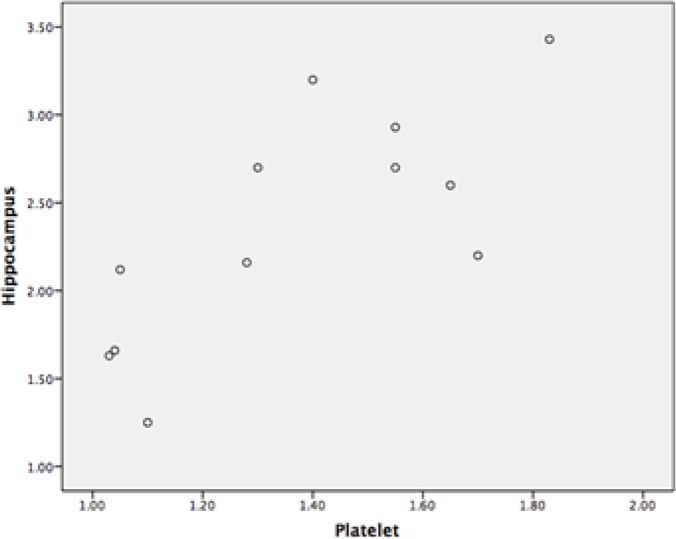
The relationship between the hippocampus and platelet in terms of PKC activity Scatterplot showed a positive Pearson correlation coefficient between the hippocampus and platelet in PKC activity.

The Pearson product-moment correlation coefficient was used to assess the relationship between the hippocampus and platelet in PKC activity of the Aβ-treated group. Our results showed a positive correlation between the hippocampus and platelet in PKC activity (P<0.75; r=0.01) (Figure 5).

### 3.4. PKCɛ signaling in the hippocampus and platelet of the treated groups using real-time qPCR

**Figure 6.**
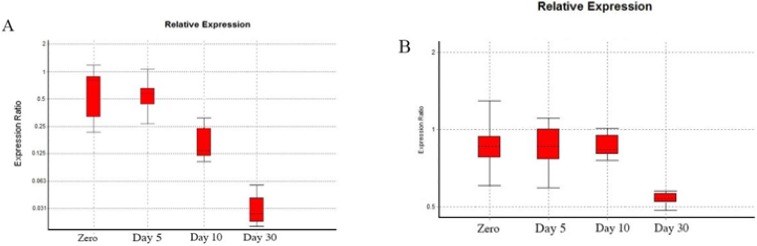
PKCɛ expression ratio assay using quantitative reverse transcription PCR at four time points (0, 5, 10, and 30 days) after Aβ injection GAPDH and β-actin were used as the reference genes to normalize the results. A. Expression ratio in the hippocampus. Aβ significantly downregulated the PKCɛ mRNA expression on day 30 (P<0.000) B. Expression ratio in platelet. No significant difference was observed between the treated and sham groups.

We studied the profile of PKCɛ expression by RT-qPCR in the hippocampus and platelet of Aβ-treated rats. The target sequence was confirmed by sequencing of PCR products. Aβ (5 μg/μL) significantly downregulated the PKCɛ mRNA expression in the hippocampus of rats on day 30 (P<0.000), but there was no significant difference in PKCɛ expression in platelet (P<0.059) (Figure 6 A & B).

### 3.5. PKCɛ signaling in the hippocampus and platelet of the treated groups using western blotting analysis

**Figure 7.**
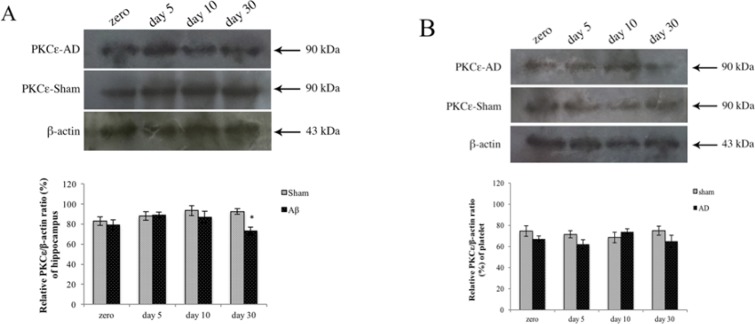
Protein expression assay of PKCɛ using western blot analysis at four time points (0, 5, 10, and 30 days) after Aβ injection Mean PKCɛ/β-actin is presented as histogram bars. A. The gel mobility of PKCɛ 90-kDa band in four groups in the hippocampus sample. PKCɛ/β- activity in the treated group reduced significantly (P<0.05) versus sham group on day 30. B. The gel mobility of PKCɛ 90-kDa band in four groups in platelet sample. No significant difference was observed between the treated and sham groups.

The effect of Aβ on PKCɛ protein expression was assessed with western blot analysis. There was no significant difference on the days 0, 5, 10, and 30 after the injection in the sham and also Aβ-treated groups using one-way ANOVA. Aβ significantly downregulated the PKCɛ protein expression in the hippocampus of treated groups on day 30 (P<0.05); however, there was no significant difference in PKCɛ expression in platelet sample (Figure 7 A & B).

## 4. Discussion

AD is a neurodegenerative disorder that affects several parts of the brain, such as the hippocampus, olfactory bulb, cortical regions, cerebellum, and hypothalamus (El Khoury, Gratuze, Papon, Bretteville, & Planel, 2014). Aβ is one of the most important factors to induce AD. It affects the hippocampus and platelet PKCɛ-load leading to memory and neurobehavioral profile impairment (Roher et al., 2009).

We aimed at finding the effect of AD on the activity and expression of PKC in reducing spatial learning and memory by examining the hippocampus and platelet of adult rats. The MWM test was employed to study spatial learning and memory. In this regard, traveled distance, escape latencies, and visual platform and probe tests (time spent in the target quadrant) were evaluated. The results showed that the traveled distance was significantly longer and escape latency was significantly shorter in Aβ-treated groups. Aβ has widely been used to create an AD model (Frid, Anisimov, & Popovic, 2007; Hoppe et al., 2013; Majlessi, Choopani, Kamalinejad, & Azizi, 2012; Prakash, Medhi, & Chopra, 2013; White, Manelli, Holmberg, Van Eldik, & Ladu, 2005); therefore, we injected Aβ (1–42) in the rat brain, to simulate a neurode-generative brain in the present study.

To examine the role of PKC in the pathophysiology of AD, we measured the total PKC activity and also PKC isoenzyme, PKCɛ, mRNA expression, and protein levels in the platelets and CA1 region of the hippocampus in the treated and sham groups. PKC activity assessment showed that its activity in the AD brain group was lower than the sham group, which was significant 10 days after Aβ injection, and this significant difference was observed 30 days after Aβ injection in platelet samples. The small obtained standard error demonstrated the reliability of this survey on subjects.

The observed reduced activity was consistent with the results of previous studies. Reportedly, the reduced activity of two PKC isoenzymes of α and ɛ is indirectly associated with enhanced Aβ levels in an AD transgenic mouse model. They also showed that PKC activators could also prohibit the amyloidogenic pathway via inhibition of the β-site of APP cleaving enzyme (Hongpaisan et al., 2011). Other studies also demonstrate that PKCɛ activity is significantly lower in the AD brain than the control group (Matsushima, Shimohama, Chachin, Taniguchi, & Kimura, 1996; Saitoh et al., 1989). Lanius R.A et al., (1997) showed that there were no significant differences in PKC activity in the frontal, motor, temporal, and parietal cortex or also in the leukocytes and platelets of AD patients and controls. In addition to the changes in PKC activity, we observed some differences in PKCɛ gene expression and also protein level in the CA1 region of the hippocampus between AD and sham groups.

One month after Aβ injection, the expression ratio of PKCɛ was significantly changed in the hippocampal tissue compared with the sham groups, but there was no significant difference in its expression in platelets. Unsworth, Smith, Gissen, Watson, & Pears (2011) reported that PKCɛ negatively regulate ADP-induced aggregation and dense granule secretion. Murugappan et al., study was conducted on platelets containing thromboxane. This lipid generates ADP, which reinforces aggregation and causes dense granule secretion in platelets (Murugappan, Shankar, & Kunapuli, 2004). Also, some studies have demonstrated that aspirin or indomethacin abolished the generation of thromboxane. Under these conditions, loss of PKCɛ in murine platelets negatively regulates intracellular calcium mobilization, which regulates thromboxane generation (Bynagari-Settipalli et al., 2012).

The insignificant difference in the PKCɛ expression in platelets may be due to their low level. Buensuceso et al., (Buensuceso et al., 2005) were unable to detect PKCɛ protein in human platelets and another study attempted to address this through concentration of PKCɛ by immunoprecipitation, which was unsuccessful, as well. They mentioned that PKCɛ is expressed at high levels in mouse but not human platelets, and also the differences in batches of antibodies and absence or existence of PKCɛ may explain the inconsistent results (Pears et al., 2008).

Quantitation of isoform-specific immunoreactivity was assessed by western blot analysis. It showed that AD significantly downregulated the PKCɛ protein expression in the hippocampus of the treated groups on day 30, but there was no significant difference in PKCɛ expression in the platelet sample. These results confirmed the expression level of PKCɛ gene using real-time PCR. These determinations have been done in several previous reports with conflicting results (Bosman, 1992; Lanius et al., 1997; Matsushima et al., 1996; Molchan et al., 1993).

Several studies also have assessed the PKC activity and protein level using quantitative autoradiography or immunological assays, like western blots in AD and sham groups. They did not find a significant difference between AD and sham tissues (Masliah et al., 1990; Shimohama et al., 1993). Western blot analysis was used to measure PKCɛ and PKCα in hippocampal neurons. For these measurements, the entire hippocampal areas were assessed (Bar-Am, Yogev-Falach, Amit, Sagi, & Youdim, 2004). Based on the reported results, subtle changes in protein levels and or compartments containing protein in distinct areas of the hippocampus were found using immunohistochemistry methods. It has also demonstrated that a decrease in PKCɛ protein level in the hippocampal CA1 region of Tg2576 mice was due to the decreased number of presynaptic terminals containing PKCɛ (Hongpaisan, Sun, & Alkon, 2011).

The intracellular production of Aβ can be a potential threat to the cells. The enhancement of Aβ concentration increases the risk of its aggregation and the occurrence of degenerative changes (Oh et al., 2005). These events are observed in transgenic mice and also in areas known to be affected by AD pathology (Chui et al., 1999; Wirths et al., 2001), such as the hippocampus (Gouras et al., 2000). Different isoforms of PKC may be involved and can also play a role in the complex regulation of APP metabolism. Many of these enzymes have been shown defective in AD (Gasparini et al., 1998). In some cases, these defects were associated with aberrant APP metabolism (Bergamaschi et al., 1995; Govoni et al., 1993; Govoni et al., 1996; Vestling et al., 1999).

## 5. Conclusion

In conclusion, the expression ratio and protein expression level of PKCɛ reduced on day 30 following the injection of Aβ in the hippocampal tissues, but no significant difference between AD and sham groups were observed in platelets that may result from the low concentration of PKCɛ or insensitive methods to show the expression ratio of the mentioned isoform. Also, in our results, PKC activity in the hippocampus decreased after day 10, but PKCɛ expression decreased on day 30, suggesting that PKC isozymes other than PKCɛ may contribute to this change.

## Ethical Considerations

### Compliance with ethical guidelines

All experiments were executed according to the Guide for the Care and Use of Laboratory Animals (National Institutes of Health Publication; No. 80-23, revised 1996).
